# Effects of the built environment on physical activity: a systematic review of longitudinal studies taking sex/gender into account

**DOI:** 10.1186/s12199-020-00915-z

**Published:** 2020-11-27

**Authors:** Antonina Tcymbal, Yolanda Demetriou, Anne Kelso, Laura Wolbring, Kathrin Wunsch, Hagen Wäsche, Alexander Woll, Anne K. Reimers

**Affiliations:** 1 Department of Sport Science and Sport, Friedrich-Alexander-University University Erlangen-Nuremberg, Gebbertstraße 123b, 91058 Erlangen, Germany; 2 Department of Sport and Health Sciences, Technical University of Munich, Munich, Germany; 3 Institute of Sports and Sports Science, Karlsruhe Institute of Technology (KIT), Karlsruhe, Germany

**Keywords:** Health equity, Active transport, Active commuting, Men, Women, Built environment, Physical activity, Gender

## Abstract

**Background:**

Individual health behavior is related to environmental and social structures. To promote physical activity (PA) effectively, it is necessary to consider structural influences. Previous research has shown the relevance of the built environment. However, sex/gender differences have yet not been considered. The aim of this systematic review was to identify built environmental determinants of PA by taking sex/gender into account.

**Methods:**

A systematic literature search was carried out using six electronic databases (PubMed, CINAHL, SportDiscus, PsycInfo, Scopus, Web of Knowledge) to identify studies analyzing the effect of changes in the built environment on PA, taking sex/gender into account. To be included, studies had to be based on quantitative data and a longitudinal study design. Changes in the built environment had to be objectively assessed. The methodological quality of the studies was examined using the QualSyst tool for examining risk of bias.

**Results:**

In total, 36 studies published since 2000 were included in this review. The data synthesis revealed that the majority of reviewed studies found the built environment to be a determinant of PA behavior for both, males and females, in a similar way. Creating a new infrastructure for walking, cycling, and public transportation showed a positive effect on PA behavior. Findings were most consistent for the availability of public transport, which was positively associated with overall PA and walking. The improvement of walking and cycling infrastructure had no effect on the overall level of PA, but it attracted more users and had a positive effect on active transportation. In women, the availability of public transport, safe cycling lanes, housing density, and the distance to daily destinations proved to be more relevant with regard to their PA behavior. In men, street network characteristics and road environment, such as intersection connectivity, local road density, and the presence of dead-end roads, were more important determinants of PA.

**Conclusion:**

This review sheds light on the relevance of the built environment on PA. By focusing on sex/gender differences, a new aspect was addressed that should be further analyzed in future research and considered by urban planners and other practitioners.

## Introduction

Currently, there is convincing evidence that physical activity (PA) plays an important role in maintaining physical and mental health [1]. Insufficient PA is one of the main risk factors for developing non-communicable diseases [2]. Moreover, insufficient PA poses a large burden on national economies [3]. Besides personal characteristics, PA of individuals is also related to contextual factors, such as environmental and social structures [4]. A recent study on the worldwide epidemiology of insufficient PA, which included data from 168 countries, estimated that 27.5% of the pooled sample did not meet the WHO recommendations on PA for health. Additionally, in 159 of 168 countries, the level of PA was lower in women than in men [5]. In this regard, it is necessary to develop and implement new interventions aiming at PA promotion that target both, males and females. By targeting males and females equally, intervention-generated inequalities in health behaviors, such as PA and resulting health benefits, can be avoided [6].

The differences in PA behavior between males and females can on the one hand be explained by sex-linked biology and on the other hand by gender socialization and social norms. Based on Johnson et al. [7], gender refers to “the socially constructed roles, behaviors, expressions and identities of girls, women, boys, men and gender diverse people.” In acknowledging the theoretical entanglement of biological factors and gendered social factors, we use the term sex/gender in the present article [8].

Besides sex/gender differences, various studies have indicated that the built environment is a relevant prerequisite for PA [9–13]. Built environments can be defined as any human-made environments that generate needs and provide opportunities for active travel and PA including transportation infrastructure, land use patterns, and urban design characteristics [14, 15]. Sallis et al. in their ecological model of four domains of PA [16] described that built environment features may also be referred to the transportation, recreation, occupation, or household domain.

In recent years, there has been a large number of studies examining which built environmental characteristics have an impact on PA behavior, with the majority of studies being cross-sectional. The longitudinal relationships should be taken into account to identify causal effects of built environmental determinants on PA. Gebel et al. [17] emphasize that controlled prospective evaluations of environmental interventions and relocation studies in which the same individuals are exposed to different environments provide the highest level of evidence in this research area. For planning built environmental interventions, it can be recommended to take into account data from studies that measured built environmental characteristics objectively. Objective measures are more appropriate as starting points for intervention programs than subjective perceptions of the environment because objective measures can be directly addressed when applying changes in the built environment. Additionally, objective measures seem to be more appropriate starting points for stopping the declining rates of PA on the population level whose underlying causes cannot be primarily ascribed to individuals and their perceptions [18]. Furthermore, objective measures have been shown to have a stronger association with walking than subjective measures [19].

In addition, the needs of diverse target groups should be taken into account when planning built environment interventions, because diverse groups live in the same neighborhoods and should benefit equally from such interventions. With regard to sex/gender, it has been shown, for example, that crime safety may be more relevant for women, while men are more sensitive to pedestrian and traffic safety [20, 21]. Taking into account the sex/gender differences in PA and its built environmental determinants as well as PA-related gender health inequalities, it is especially important to address both, males and females concomitantly when planning intervention studies. Despite the fact that the influence of built environment on PA behavior is currently of interest in many studies and reviews [9–13], a systematic analysis of whether the influence of the built environment is equal for/differs between males and females is not available.

Thus, the objective of the present systematic literature review is to identify built environmental determinants of PA by taking sex/gender into account. This is an important precondition to identify starting points of built environmental interventions and to create effective approaches aiming at PA promotion in both, males and females.

## Methods

The review follows the guidelines of the PRISMA (Preferred Reporting Items for Systematic reviews and Meta-Analyses) statement [22]. The protocol was registered to the international prospective register of systematic reviews PROSPERO (www.crd.york.ac.uk) on July 14, 2020 with registration number CRD42020169923.

### Eligibility criteria

Articles were included if they reported a quantitative empirical study with a longitudinal pre-post-design (i.e., prospective longitudinal cohort studies or natural experiments with at least two data collection points, including repeated cross-sectional surveys) investigating the relationship between built environmental features and PA taking sex/gender in statistical analyses and/or presentation of results into account. Qualitative studies, cross-sectional studies, or those that did not consider sex/gender were excluded. Additionally, studies examining populations with specific health impairments such as overweight and obesity or cognitive and psychological disorders were also excluded. Only healthy participants representing the general population without any age restriction were eligible.

For the purpose of this review, “built environment” was broadly defined as any human-made environment that generates needs and provides opportunities for active travel and PA [14, 15]. A change in the built environment had to be objectively assessed using geographical information systems (GIS), desktop mapping, or other audit tools. Natural experiments should be focused on changes in the built environment in terms of moving from one kind of environment into another or changes in infrastructure (creating new walking or cycling trails, park improvements, etc.). This inclusion criterion is based on the aim to identify starting points for built environmental interventions to improve PA. Hence, studies including perceived measures of the built environment were excluded. In addition, studies that focused solely on neighborhood socioeconomic status or socio-environmental aspects (including population density) were also excluded.

Changes in PA behavior were used as the primary outcome. All possible types of PA outcome were eligible including overall PA, moderate to vigorous physical activity (MVPA), exercise, walking, steps per day, cycling, jogging, active transport, number of cyclists, and number of visitors of PA facilities. Studies with both objective and subjective (self-reported) assessments of PA were also included.

All included articles had to be written in English language and published in scientific journals. In order to track the most current trends, it was decided to limit the search to articles that were published from January 2000 onwards and listed in the databases up to March 12 2020.

### Information sources and search strategy

A systematic search in the databases PubMed (Ovid), CINAHL (EBSCO), SportDiscus (EBSCO), PsycInfo (EBSCO), Scopus (Elsevier), and Web of Knowledge was conducted on March 12, 2020. Additionally, reference lists from previous reviews on associations between built environment and PA and other relevant publications were screened to identify other potentially eligible studies. A comprehensive search strategy was developed using the SPIDER approach [23] with a combination of keywords in the categories study sample, phenomenon of interest, design, and evaluation. The search formula was as follows: (child* OR youth* OR adolescen* OR boy* OR girl* OR wom*n OR m*n OR adult* OR elderly OR aged OR student) AND (“built environment*” OR neighborhood* OR neighbourhood* OR “city planning*” OR “urban planning” OR “residence characteristic*” OR walkabil* OR bikeabil*) AND (longitud* OR follow-up OR intervention* OR experiment*) AND (“physical activ*” OR exercise OR sport* OR bicycl* OR cycl* OR “active transport*” OR “active commut*” OR “active travel” OR walk*).

### Study selection

Two reviewers (AT and SF) independently screened and selected the relevant articles. First, all articles were screened based on titles and abstracts. In a second step, full texts of potentially relevant articles were reviewed. If necessary, supplementary files were also reviewed for additional information. Disagreements between the reviewers were discussed until a consensus was reached.

Records were managed in Covidence systematic review software (Veritas Health Innovation, Melbourne, Australia; www.covidence.org) and EndNote x9 (Clarivate Analytics, Philadelphia, PA, USA).

### Data extraction

Data of included studies were extracted and summarized by one researcher, with verification by another reviewer, in order to reduce bias and error. Extraction included the following items: general study information (authors, year of publication, country), description of study sample (age, gender, number of participants), study design, follow-up time, objective built environment variables, PA variables, statistical analysis, and overall and gender specific results on associations between changes in built environment and PA (see Table 1).

**Table 1:** Study characteristics

Study details	Participant characteristics (% female)	Follow-up time	Types of built environment, measurement instrument/description of intervention	PA outcome, (S—subjective measurement; O—objective measurement)	Statistical analysis	Results on associations between changes in BE and PA		Quality score
						Overall	By gender	
Prospective longitudinal cohort studies								
Boone-Heinonen et al. 2010, USA	Adolescents and young adults *N* = 12,701 (50.9%)	6 years	Pay and public PA facilities availability (count per 10,000 population) Street connectivity (Alpha index) Landscape diversity (Simpson’s diversity index)	Leisure time MVPA (S)	Fixed effects Poisson regression	In the whole sample landscape diversity, public facility availability, and alpha index were unrelated to MVPA.	Leisure-time MVPA bouts associated with increased public facility availability among female movers (0.053, 95% CI: 0.008, 0.097) and with pay facility availability among men (0.024, 95% CI: 0.006, 0.042, *p* < 0.05) No significant gender specific effects of landscape diversity and street connectivity on leisure-time MVPA bouts.	1
Buck et al 2019, Germany, Italy, and Sweden	Children and adolescents 3–15 years old *N* = 2488 (48.1%)	6 years	Movability index (land use mix, street connectivity, availability of public transport and public open spaces)	LPA and MVPA (O)	Linear mixed model	Results presented separately for genders.	In girls, the movability index showed a consistent significantly positive effect on MVPA (β = 2.14, 95% CI: (0.11; 4.16)) for all ages, and in boys, on LPA with age for each year (β = 2.68, 95% CI: (0.46; 4.90)). Availability of public open spaces was more relevant for MVPA in girls (β = 2.38, 95% CI: (0.43; 4.34)) and LPA in boys (β = 10.6, 95% CI: (4.78; 16.3)) during childhood, whereas in adolescence, intersection density (β = 3.36, 95% CI: (1.14; 5.57)) became more important for boys LPA.	0.86
Carver et al. 2010, Australia	Children 8–9 years old *N* = 170 (49%) Adolescents 13–15 years old *N* = 276 (57%)	2 years	Road environment; GIS	MVPA (O) Active transport (walking cycling) (S)	Linear regression	Results presented separately for genders.	Children Boys: Change in MVPA was positively associated with the number of slow points (chicanes) in the neighborhood (β = 1.55, 95% CI = 0.25 to 2.86) (before school), the total length of locals roads (B = 3.81, 95% CI = 0.95 to 6.67) (weekends) and intersection density (B = 0.49, 95% CI = 0.14 to 0.84) (weekends). Girls: Change in MVPA was negatively associated with the intersection density (B = − 0.05, 95% CI = − 0.09 to − 0.003) and the number of traffic/pedestrian lights (B = − 0.88, 95% CI = − 1.41 to − 0.35) (evenings). Adolescents. Boys: The number of speed humps in the neighborhood was positively associated with change in MVPA after school (*p* < 0.05), and the local road index was negatively associated with change in MVPA on weekend days (*p* < 0.05). Girls: Total length of local roads (*p* < 0.01), intersection density (*p* < 0.01), and the number of traffic/pedestrian lights (*p* < 0.05) were each positively associated with change in MVPA before school. The local road index was negatively associated with change in MVPA after school (*p* < 0.01). The numbers of speed humps (*p* < 0.05) and gates/barriers (*p* < 0.01) were positively associated with change in MVPA during evenings. The total length of local roads (*p* < 0.01), intersection density (*p* < 0.05), and the number of speed humps (*p* < 0.05) were positively associated with change in MVPA during non-school hours, while the local road index was negatively associated with MVPA (*p* < 0.01). The number of speed humps was positively associated with change in MVPA on weekend days (*p* < 0.05).	0.82
Coogan et al. 2009, USA	Adult women *N* = 20,354 (100%)	6 years	Housing density, land use, street connectivity, traffic, public transit availability, presence of sidewalks, distance to parks; GIS	Utilitarian and exercise walking (S)	Multinomial logistic regression generalized estimating equation model	(Only females)	Increases in utilitarian walking were associated with increased housing density (OR = 2.72, 95% CI 2.22, 3.31) and bus availability (OR = 1.44, 95% CI: 1.21, 1.72). Increased housing density led to increased exercise walking (OR = 1.28, 95% CI: 1.07, 1.52) Land use, street connectivity, traffic, presence of sidewalks, and distance to parks were not associated with utilitarian or exercise walking.	0.91
Coombes et al. 2014, UK	Children 10–11 years old *N* = 518 (55.8%)	1 year	Availability of greener environments and destinations, density of the road network, school-home distance; GIS	Overall PA (O). Travel mode (S)	Multiple regression models	No significant associations between change in school commute environment or home neighborhood supportiveness and overall PA.	No gender specific effects.	0.82
Crawford et al. 2010, Australia	Children *N* = 301 (57.5%)	5 years	Destinations (PA related and school), road connectivity, traffic exposure; GIS.	MVPA (O)	Generalized estimating equation	Results presented separately for genders.	No significant associations between BE and MVPA, only the presence of dead-end roads was positively associated with MVPA in boys (*b* = 0.007, 95% CI: 0.01, 0.13; *p* < 0.05).	0.86
Dowda et al. 2020, USA	Children *N* = 555 (56%)	3 years	Outdoor PA equipment	Number of PA days and PA location (S)	Longitudinal Poisson regression	Outside PA equipment were positive significant predictors of street PA in total sample (*p* < 0.05).	No gender specific effects.	0.82
Evenson et al. 2018, USA	Adolescents girls *N* = 265	1 year	Parks availability; GIS	Park visits; MVPA in parks (O)	Wilcoxon sign rank test for two dependent samples, Pearson correlation coefficients	(Only females)	Parks were an under-used resource for adolescent girls, particularly for MVPA. Only one-fifth of the sample (20% at baseline, 19% at follow-up) visited a park at least once in six days of observation. The average duration of park visits was higher at baseline (63.9 min) compared to follow-up (38.4 min). On days when a park was visited, MVPA was higher than on days when a park was not visited. However, only 1.9% (baseline) and 2.8% (follow-up) of MVPA occurred in parks.	0.77
Hou et al. 2010, USA	Adults *N* = 5115 (54.5%)	15 years	Street connectivity (intersection density, link-node ratio) , characteristic of local roads (density, proportion local relative to total road); StreetMap data, TIGER/line road classification	Overall walking, cycling, and jogging (S)	Two-part marginal effect model (probit model and an ordinary least squares regression model)	Results presented separately for genders.	Intersection density was positively associated with walking, bicycling and jogging frequencies in low urbanicity areas for both genders (men: β = 1.0; 95% CI: 0.04, 1.9, *p* = 0.04; women: β = 1.3; 95% CI: 0.6, 2.0, *p* = 0.001). Density of local roads was positively associated only among men in low urbanicity areas (β = 1.0; 95% CI: 0.1, 2.0, *p* = 0.03). In high urbanicity areas walking, cycling and jogging frequencies in women were negatively associated with local road density (β = − 1.3; 95% CI: − 2.2, − 0.3, *p* = 0.007) and proportion of local roads (β = − 1.4; 95% CI: − 2.3, − 0.6, *p* = 0.001). No significant associations between link-note ratio and PA for both genders.	0.86
Michael et al. 2010, USA	Adult men (> 65 years old) *N* = 513	Average 3.6 years	Availability of proximate PA resources: parks, trails, and recreational facilities; GIS	Walking (S)	Log-binomial regression	(Only males)	Proximity to recreational facilities was not associated with walking. Distances to a park and a trail were positively associated with maintaining or increasing walking between baseline and follow-up, but was not significant for the whole sample. Proximity to parks and proximity to trails, respectively, were associated with a 22% (95% CI: 1.01, 1.47) and 34% (95% CI: 1.16, 1.55) higher likelihood of maintaining or increasing walking time in high-SES neighborhoods, but there was no association in low-SES neighborhoods.	0.77
Sanders et al. 2015, Australia	Children *N* = 4983 (49.1%)	8 years	Availability of green areas	Overall PA (S)	Multilevel linear regression and multilevel logistic models	Results presented separately for genders.	Boys living in areas with 10 % more neighbourhood green space had a 7 % (95% CI = 1.02, 1.13) greater odd of choosing physically active pastimes; and 7% (95% CI = 1.02; 1.12) and 9% (95% CI = 1.03; 1.15) greater odds of meeting PA guidelines on weekdays and weekends, respectively. A 10% difference in green space was associated with a mean of 1.9 min greater time spent physically active on a weekday (β = 1.88, 95% CI = 0.22, 3.53; *p* = 0.026), and 3.0 min more weekend PA (β = 3.01, 95% CI = 0.37, 5.66; *p* = 0.026) after adjusting for confounders, but only at younger ages. No statistically significant results were observed for girls.	0.91
Schipperijn et al. 2015, Denmark	Young adults, *N* = 177 (57%)	6 years	Movability index, recreational facilities, density of daily destinations, street connectivity; GIS	Overall PA (O)	Multivariable analysis of variance	No significant associations between changes in movability index, availability in recreational facilities, density of daily destinations, street connectivity and PA for the whole sample.	Increases in mean daily total PA associated with increases in movability index (β = 10.15, 95% CI: 2.08, 18.21, *p* = 0.014) and daily destinations (β = 31.24, 95% CI: 10.64, 51.84, *p* = 0.003) in females. Increased intersection density (street connectivity) was negatively associated with mean daily total PA in males (β = − 35.47, 95% CI: − 67.10, − 3.83, *p* = 0.03)	0.91
Intervention studies								
Andersen et al. 2017, Denmark	Adolescents *N*_baseline_ = 354 (53%) *N*_post-renewal_ = 319 (59.6%)	1 year	New urban green spaces and playgrounds were created	PA within the renovated area (O).	Linear mixed model	Post-intervention sample spent 7.8 min per day in LPA (*p* = 0.012) and 4.5 min per day in MPVA (*p* < 0.001).	No gender specific effects.	0.77
Brown and Werner 2007, USA	Adults *N* = 51 (47%)	1 year	New rail stop	Moderate PA bouts (O).	Ordinary least squares regression	Rail ridership was positively associated with moderate activity bouts, β = 0.39 (SE = 0.01), *p* = 0.01.	No gender specific effects.	0.77
Burbidge and Goulias 2009, USA	Children and adults from 5 years old *N* = 82 (54.9%)	1 and 5 months	New trail	Overall PA, walking and biking trips (S)	A fixed-effects panel analysis regression	The new trail was associated with significant decrease in total PA (-0.245, *t* value = − 2.13, R-square = 0.045, *p* = 0.036) and in the number of walking trips (− 0.265, *t* value = − 2.71, R-square = 0.070, *p* = 0.008).	No gender specific effects.	0.77
Chang et al. 2017, Mexico	Adults *N*_pre-intervention_ = 1067 (52%) *N*_post-intervention_ = 1420 (51%)	3 years	Bus rapid transit and streetscape redesign (widened sidewalks, road diets, recovery of green and public space)	Walking for transport, walking for recreation, and cycling for transport (S)	Propensity score matching, cluster analysis	The average treatment effect of living in post-intervention neighbourhood on walking for transport was 24.37 min per week, on walking for transport and recreation—31.72 min, on cycling for transport—4.81 min.	Cluster analyses showed that females with low education experienced the greatest increases in PA. All of the female clusters experienced significant growth in recreational walking and transport walking except female homemakers with high education. Male clusters experienced either minimal improvements in recreational walking or decreases, but significant improvement in walking for transport (the greatest increase in male students with mid-level education).	0.82
Cohen et al. 2015, USA	*N*_baseline_ = 922 *N*_post-renewal_ = 1043	3 years	Park improvements	Park use and PA (O)	Mixed effect model and logit models	Use of the two renovated parks and PA level of users increased compared with baseline. %-change in total park use (β = 233.1, SE = 55.9, *p* = 0.001); %-change in MET-hours expended in park (β = 254.8, SE = 70.1, *p* = 0.001). The total park use and MET-hours expended in unrenovated parks significantly decreased.	No gender specific effects.	0.86
Cohen et al 2014, USA	N/A	2 years	Creation of pocket parks	Park use and PA (O) Self-reported park use	Generalized estimating equation	The new pocket parks had significantly more users than comparison park playgrounds (β = − 1.21, SE = 0.28, *p* = 0.001).	More females were observed at the pocket parks during follow-up than at comparison park playgrounds (63% vs. 56%, *p* = 0.0068). Females were somewhat less active than males in the pocket parks, with 22% engaged in MVPA vs. 29% of males (*p* = 0.08).	0.68
Cranney et al. 2016, Australia	Children and adults *N*_baseline_ = 8560 (46%) *N*_post-installation_ = 7097 (46%) *N*_follow-up_ = 8248 (46%)	1 year	Park improvement (outdoor gym)	Park use and PA (O)	Two sample z-test	The proportion of all park users engaged in MVPA increased significantly (*p* < 0.001) from baseline (9.4%) to post-installation (12.8), then decreased toward the baseline proportion at follow-up. The proportion of outdoor area users from all park users increased from 2.4% to 6% (*p* < 0.001) and decreased back to 3.3% at follow-up, which is still a significant increase in comparison with baseline (*p* < 0.001).	The proportion of male park users engaged in MVPA during follow-up measurement increased on 1.9% in comparison with baseline (*p* < 0.01), for female users increase was not significant. The proportion of male outdoor area users from all park users during follow-up measurement increased on 1.1% in comparison with baseline (*p* < 0.01), for female users increase was not significant.	0.77
Dill et al. 2014, USA	Adults *N* = 353	2 years	New bicycle boulevard	MVPA (O). Walking and cycling trips (S)	Binomial logit regression, negative binomial regression and linear regression models	No significant associations between installation of bicycle boulevards and increases in PA and active transportation.	Women engaged in less MVPA (ß = − 4.46, *p* = 0.02) and minutes of bicycling (ß = − 0.311, *p* = 0.01), were less likely to cycle more than 10 min (ß = − 0.475, *p* = 0.04) and make bike trips (ß = − 0.58, *p* = 0.01), but were more likely to walk more than 20 min (ß = 0.616, *p* < 0.001).	0.85
Goodman et al. 2013, UK	Adults *N* = 1510 (57%)	2 years	New local walking and cycling routes	Walking and cycling at new routs (S)	Longitudinal Poisson regression	After one year 32% of sample started to use new routes (29% walking, 13% cycling), after two years the proportion of users increased to 38% (35% walking, 16% cycling).	Men were more likely to use Connect2 (rate ratio 1.14 for men vs. women, *p* < 0.05). However, results were significant only in one of three cities.	0.91
Heien et al. 2015, UK	Adults *N* = 470 (66,6%)	3 years	New transport infrastructure (busway with path for walking and cycling)	Commute mode share (active travel) (S)	Multivariable multinomial logistic regression models	Commuters living 4 km from the busway were almost twice as likely to report a substantial increase (> 30%) in their active travel mode share (relative risk ratio [RRR] 1.80, 95% confidence interval [95% CI] 1.27 to 2.55), and half as likely to report a small decrease RRR 0.47, 95 % CI 0.28 to 0.81), than those living 9 km away. Proximity to the busway also predicted a large decrease in the share of trips made entirely by car (RRR 2.09, 95 % CI 1.35, 3.21).	No gender specific effects.	0.86
Heinen et al. 2018, Australia	Adults *N* = 4637 (57.7%)	4 years	New public bicycle-sharing scheme	Time spent cycling (S)	Multinomial logistic regression	On average, the respondents decreased the total time spent cycling by 1.98 minutes a week. Time spent cycling for transport decreased by 2.34 min per week, whereas the average time spent cycling or recreation increased by 0.35 min. No significant associations between proximity to a bicycle-sharing station and changes in time spent cycling.	Women, when compared to men, were less likely to increase or decrease the time spent cycling.	0.82
King et al. 2015, USA	Children and adults *N*_baseline_ = 2888 (42.2%) *N*_post-intervention_ = 4525 (46.4%)	2 years	New park	Park use and PA (O)	T-test	The total number of people observed using the park post-construction significantly increased (*p* = 0.004). Three-fold increase in energy expended within the park after construction (*p* = 0.002). Increase in the proportion of park users who were engaged in moderate (*p* = 0.007) or vigorous activity (*p* = 0.04).	The number of visitors increased for both genders. Proportion of visitors engaged only in sedentary activities decreased both in females (from 59 to 42%) and in males (from 44 to 26%). Proportion of females observed engaging in vigorous PA inside of the park boundaries increased from 0 to 20% (mostly children). Adolescent females were very under-represented within the park. Of the adolescent females counted, few were engaged in vigorous PA. On the other hand, there was a significant increase in the proportion of adolescent males observed engaging in vigorous PA (*P* < 0.001), especially in playfields.	0.77
Ng et al. 2020, Australia	Preschoolers 2-5 years old *N*_baseline_ = 223 *N*_follow-up_ = 116	6–12 months	Upgrade od childcare outdoor spaces (installation of outdoor PA equipment)	Overall PA, MVPA (O)	Multivariable linear regression	Intervention preschoolers were more active than control at follow-up (58.09 vs. 42.13 min/day increase in total PA; 30.46 vs. 19.16 min/day increase in MVPA (all *p* < 0.001)).	Boys were significantly more active than girls (*p* < 0.01).	0.86
Panter et al. 2017, UK	Adults *N* = 1257	2 years	New walking and cycling infrastructure	Walking for transport and recreation (S)	Latent class analysis and multinomial regression	Short-lived and sustained increase as well as uptake in walking for transport and recreation were associated with use of new walking and cycling infrastructure. Proximity to the intervention was associated with both uptake of and short-lived increases in walking for transport.	Increase and uptake in walking for transport or recreation were not associated with gender.	0.82
Parker et al. 2011, USA	Adults N/A	1 year	New bike lane	Number of cyclists (O)	Negative binomial regression	The mean number of cyclists observed per day increased by 57% (*p* < 0.001).	The increase among adult female riders (133%, *p* < 0.001) was greater than among adult male riders (44%, *p* < 0.001).	0.6
Parker et al. 2013, USA	Youth and adults N/A	1 year	New bike lane	Number of cyclists (O)	Negative binomial regression	There was an increase in cyclists on all three streets after the installation of the bike lanes [62.5 (± 28.8) vs. 110 (± 109); *Z* = 8.97, *p* < 0.000], with the largest increase on the street with the new lane [pre 79.2 (± 30.5), post 257.1 (± 50.9); *Z* = 10.79, *p* < 0.000].	The increase in cyclists was greater among females (4.69) than males (3.12).	0.75
Rissel et al. 2015, Australia	Adults *N* = 512 (62.5%)	1 year	New cycling infrastructure	Number of cyclist (O). Cycling behavior (S)	Mixed-effects logistic regression models	Bike counts at two sites on the new bicycle path reported an increase of 23 % and 97 % respectively at 12 months. Weekly frequency of cycling remained higher in the intervention (29.2–25.8% at follow-up) area than the comparison area (22.4–23.2% at follow-up) (*p* = 0.04). Among the participants in the cohort, there was no change in the self-reported weekly frequency of cycling. Only 15 % participants reported using the new bicycle path, with most users (76 %) living in the intervention area.	No gender specific effects.	0.91
Schultz et al. 2017, USA	Children and adults *N*_baseline_ = 2080 (46%) *N*_1 year_ = 2275 (45%) *N*_2 years_ = 2276 (46%)	2 years	Improved access to the park	Park use and PA (S)	One-way ANCOVA model and Sidak post-hoc comparisons	Total park use increased from 2012 (*n* = 2080) to 2013 (*n* = 2275) and remained constant in 2014 (*n* = 2276). However, despite increases in safe access and overall park use, there was a significant decrease in total energy expenditure following the installation of the crosswalk that was sustained in 2014.	Male park use increased from 2012 to 2013 (6.95 to 10.49) but significantly decreased from 2013 to 2014 (10.49 to 7.82); however, there was still a significant increase from 2012 to 2014 (6.95 to 7.82). Females also showed a significant increase of park use from 2012 to 2013 (7.45 to 9.78); however, unlike males, the increased use was maintained in 2014 (9.63). The total energy expenditure both for males and females in 2014 was significantly lower than in 2012.	0.91
Smith et al. 2019, Australia	Adults *N* = 389 (58.4%)	1 year	New recreational infrastructure Peninsula Aquatic and Recreation Centre (PARC)	Use of PARC, MVPA (S)	Multivariable logistic regression	After 12 months 17,5% of sample reported occasional use of PARC and 8.7% used it on regular basis. PARC users were not significantly more likely than non-users to show improvements in their level of leisure-time PA over 12 months.	Females used PARC more often (odds ratio 2.30, 95% CI: 1.37–3.87) than males.	0.82
Sun et al. 2014, China	Young adults, *N* = 169 (56%)	10 months (3 after intervention)	Increase in land use, street connectivity, and bus accessibility	Walking behavior (S)	Multivariable linear regression model	Intervention had positive effect on walking distance and walking ratio. An increase in pedestrian network connectivity (road intersections) positively predicted walking distance (*p* < 0.001) and walking ratio (*p* < 0.001). An increased exposure to life area buildings and an increase in population density were associated with longer walks (*p* < 0.001). At the lower campus level, an increase in the number of work area buildings was associated with a decrease in subjects’ altitude ranges (*p* < 0.001), while the increased bus service resulted in more people frequenting higher elevation levels (*p* = 0.01), and a change in middle-class bus service was inversely associated with subjects’ predicted movement across hilly terrain (*p* = 0.02).	No gender specific effects.	0.82
Tannis et al. 2019, USA	Adults *N* = 88 (78.4%)	1 year	Move into houses with active design (more attractive stairwells, outdoor community garden area, outdoor fitness area, community gym)	PA (S) Steps per day (O)	T-tests and Mann–Whitney *U* test	The greater daily steps increase had AD residents who moved from an elevator building (*p* = 0.051). Difference in MVPA between AD and non-AD residents was not significant.	AD building women reported more work-related MVPA overall (*p* = 0.01). AD men engaged in more moderate recreational PA (*p* = 0.044).	0.91
Tester and Baker 2009, USA	Children and adults N/A	1 year	Park improvements (playfields)	Park use and PA (O)	*T* test	Results presented separately for genders.	There were significant increases of the amount of playfield users among children and adults of both genders at the intervention parks (*p* < 0.05), but not in the control park. There were statistically significant increases among males and females who were observed at each respective PA level (sedentary, moderate, vigorous) in the intervention parks (p<0.05). On the control playfield, only moderately active males increased.	0.75
Wells and Yang 2008, USA	Adult women *N* = 32 (100%)	4 years	Moving to neo-traditional neighbourhood	Overall walking (O)	Mixed modeling	(only females)	Women who moved to places with fewer cul-de-sac, on average, walked more (5303 more steps per week, or 757 more steps per day, *p* = 0.025). Increases in land-use mix were associated with less walking (31,820 fewer steps per week, or 4545 fewer steps per day), *p* = 0.013.	0.86
West and Shores 2015, US	Adults *N* = 273 (41.1%)	1 year	New greenway	Walking, MVPA (S)	Ordinary least squares regressions	No significant differences between the experimental and control groups in days of walking, moderate activity, or vigorous activity before and after the greenway was constructed.	No gender specific effects.	0.88

*N* number of participants, *PA* physical activity, *LPA* light physical activity, *MVPA* moderate to vigorous physical activity, *AD* active design

### Risk of bias assessment

The QualSyst tool developed by the Alberta Heritage Foundation for Medical Research was used to assess the methodological quality of each study [24]. The tool was selected as it allows to evaluate and compare studies with different designs. Studies were evaluated according to 14 criteria, including objective, study design, method of subject/comparison group selection, subject characteristics, intervention allocation, blinding, outcome measure definition, exposure measure definition, sample size, analytic methods, estimate of variance, control for confounding, reporting results, and conclusions. Depending on the degree to which the specific criteria were met, each item was scored as “yes” = 2, “partial” = 1, “no” = 0, and the total sum was calculated as (number of “yes” * 2) + (number of “partials” * 1). If an item was not applicable to the study, it was marked “N/A” and excluded from the total possible sum, which was calculated as 28—(number of “N/A” * 2). The summary score was calculated as a total sum divided by total possible sum. The summary score (range 0–1) indicated the risk of bias, with a higher score indicating higher quality.

### Data synthesis and analyses

The association between changes in the built environment and changes in PA was used as the primary measure effect. The data was summarized narratively due to the heterogeneity of studies and the variety of exposure and outcome measures, which prevented a quantitative meta-analysis. Built environment characteristics were classified according to the ecological model of four domains of PA [16] as related to the transportation, recreation, occupation, or household domain. Type of PA behavior outcomes were grouped in (1) PA measured in minutes or days per week, or METs; (2) walking or cycling measured in minutes or days per week, number of trips or steps per day; and (3) visitation or use of PA settings measured in count of users or time spent in location. Table 2 presents the results according to the number of associations coded as positively significant (“+”), negatively significant (“−”), or having no association (“0”). Associations with *p* value ≤ .05 were considered statistically significant. If the study indicated that built environmental characteristics affect PA in both sexes in a similar way, the mark (“+”) or (“−”) was placed in the column “All.” In case the effect was observed in only one of the sexes, the mark (“+”) or (“−”) was placed in the column “Female” or “Male,” respectively.

**Table 2:** Key findings for impact of built environment on PA behavior

Domain	BE characteristics	Physical activity (minutes, days per week, METs)			Walking, cycling (minutes, days per week, number of trips, steps per day)			Visitation or use of settings (count of users, time spent in locations)		
		All	Female	Male	All	Female	Male	All	Female	Male
Transport	New walking/cycling infrastructure	0(1) 0(2) −-3)			+, f<m(4) +, f>m(5) +(6) +(7) +(8) 0(1) 0(2) 0(9) −-3)			+(9) +, f>m(10) +, f>m(11)		
	Street network characteristics, (street connectivity, road environment)	+(12) +(13) 0(14) 0(15) 0(16)	0(17)	+(18) +(16) −-17)	+(19)	0(20)				
	Local road density	−-12)	−-13)	+(13)						
	Land use mix					0(20) −-21)				
	House density					+(20)				
	Availability of public transport	+(22)			+,f>m(5) +(6) +(8) +(19)	+(20)				
	Landscape diversity	0(14)								
	Movability index	+(18)	+(17)	0(17)						
	Distance to school and daily destinations	0(15) 0(16)	+(17)	0(17)						
Recreation	Park/ green space improvement	+(23) +(24) −-25)		+(26)				+(23) +(25) +(24)		+(26)
	New parks/ green spaces	+(27) +, f<m(28) +(29)						+, f>m(28) +(29)		
	New PA facilities	0(30)						+, f>m(30)		
	Availability of PA and recreation facilities and public open spaces	+(14) +(18) 0(15) 0(17)	0^(parks)^(31) 0^(parks)^(32)	+^(parks)^(32)			+(33)			
	Outdoor PA equipment	+(34) +, f<m(35)								
Household	Houses with active design	+(36)			+(36)					

+ positive association

− negative association

0 no significant associations

f>m effect for females was greater than for males

f<m effect for females was smaller than for males

1. Dill J, McNeil N, Broach J, Ma L. Bicycle boulevards and changes in physical activity and active transportation: Findings from a natural experiment. Preventive Medicine. 2014;69:S74-S8.

2. West ST, Shores KA. Does building a greenway promote physical activity among proximate residents? Journal of Physical Activity & Health. 2015;12(1):52-7.

3. Burbidge SK, Goulias KG. Evaluating the impact of neighborhood trail development on active travel behavior and overall physical activity of suburban residents. 2009. p. 78-86.

4. Goodman A, Sahlqvist S, Ogilvie D, iConnect c. Who uses new walking and cycling infrastructure and how? Longitudinal results from the UK iConnect study. Preventive medicine. 2013;57(5):518-24.

5. Chang A, Miranda-Moreno L, Cao J, Welle B. The effect of BRT implementation and streetscape redesign on physical activity: a case study of Mexico City. Transportation Research Part a-Policy and Practice. 2017;100:337-47.

6. Heinen E, Kamruzzaman M, Turrell G. The public bicycle-sharing scheme in Brisbane, Australia: evaluating the influence of its introduction on changes in time spent cycling amongst a middle- and older-age population. Journal of Transport & Health. 2018;10:56-73.

7. Panter J, Ogilvie D, iConnect C. Can environmental improvement change the population distribution of walking? Journal of Epidemiology and Community Health. 2017;71(6):528-35.

8. Heinen E, Panter J, Mackett R, Ogilvie D. Changes in mode of travel to work: a natural experimental study of new transport infrastructure. International Journal of Behavioral Nutrition and Physical Activity. 2015;12.

9. Rissel C, Greaves S, Li Ming W, Crane M, Standen C. Use of and short-term impacts of new cycling infrastructure in inner-Sydney, Australia: a quasi-experimental design. International Journal of Behavioral Nutrition & Physical Activity. 2015;12:1-8.

10. Parker KM, Gustat J, Rice JC. Installation of bicycle lanes and increased ridership in an urban, mixed-income setting in New Orleans, Louisiana. J Phys Act Health. 2011;8 Suppl 1:S98-S102.

11. Parker KM, Rice J, Gustat J, Ruley J, Spriggs A, Johnson C. Effect of bike lane infrastructure improvements on ridership in one New Orleans neighborhood. Ann Behav Med. 2013;45(SUPPL.1):S101-S7.

12. Carver A, Timperio A, Hesketh K, Crawford D. Are safety-related features of the road environment associated with smaller declines in physical activity among youth? Journal of urban health : bulletin of the New York Academy of Medicine. 2010;87(1):29-43.

13. Hou N, Popkin BM, Jacobs DR, Jr., Song Y, Guilkey D, Lewis CE, et al. Longitudinal associations between neighborhood-level street network with walking, bicycling, and jogging: the CARDIA study. Health & place. 2010;16(6):1206-15.

14. Boone-Heinonen J, Guilkey DK, Evenson KR, Gordon-Larsen P. Residential self-selection bias in the estimation of built environment effects on physical activity between adolescence and young adulthood. The international journal of behavioral nutrition and physical activity. 2010;7:70-.

15. Coombes E, Jones A, Page A, Cooper AR. Is change in environmental supportiveness between primary and secondary school associated with a decline in children's physical activity levels? Health Place. 2014;29:171-8.

16. Crawford D, Cleland V, Timperio A, Salmon J, Andrianopoulos N, Roberts R, et al. The longitudinal influence of home and neighbourhood environments on children's body mass index and physical activity over 5 years: the CLAN study. International journal of obesity (2005). 2010;34(7):1177-87.

17. Schipperijn J, Ried-Larsen M, Nielsen MS, Holdt AF, Grøntved A, Ersbøll AK, et al. A longitudinal study of objectively measured built environment as determinant of physical activity in young adults: The European Youth Heart Study. Journal of physical activity & health. 2015;12(7):909-14.

18. Buck C, Eiben G, Lauria F, Konstabel K, Page A, Ahrens W, et al. Urban Moveability and physical activity in children: longitudinal results from the IDEFICS and I.Family cohort. The international journal of behavioral nutrition and physical activity. 2019;16(1):128-.

19. Sun G, Oreskovic NM, Lin H. How do changes to the built environment influence walking behaviors? A longitudinal study within a university campus in Hong Kong. Int J Health Geogr. 2014;13.

20. Coogan PF, White LF, Adler TJ, Hathaway KM, Palmer JR, Rosenberg L. Prospective study of urban form and physical activity in the Black Women's Health Study. American journal of epidemiology. 2009;170(9):1105-17.

21. Wells NM, Yang Y. Neighborhood design and walking. A quasi-experimental longitudinal study. American journal of preventive medicine. 2008;34(4):313-9.

22. Brown BB, Werner CM. A New Rail Stop. Tracking Moderate Physical Activity Bouts and Ridership. Am J Prev Med. 2007;33(4):306-9.

23. Cohen DA, Han B, Isacoff J, Shulaker B, Williamson S, Marsh T, et al. Impact of park renovations on park use and park-based physical activity. Journal of Physical Activity & Health. 2015;12(2):289-95.

24. Tester J, Baker R. Making the playfields even: evaluating the impact of an environmental intervention on park use and physical activity. Preventive medicine. 2009;48(4):316-20.

25. Schultz CL, Stanis SAW, Sayers SP, Thombs LA, Thomas IM. A longitudinal examination of improved access on park use and physical activity in a low-income and majority African American neighborhood park. Preventive Medicine. 2017;95:S95-S100.

26. Cranney L, Phongsavan P, Kariuki M, Stride V, Scott A, Hua M, et al. Impact of an outdoor gym on park users' physical activity: A natural experiment. Health & Place. 2016;37:26-34.

27. Andersen HB, Christiansen LB, Klinker CD, Ersbøll AK, Troelsen J, Kerr J, et al. Increases in use and activity due to urban renewal: effect of a natural experiment. American journal of preventive medicine. 2017;53(3):e81-e7.

28. Cohen DA, Marsh T, Williamson S, Han B, Derose KP, Golinelli D, et al. The potential for pocket parks to increase physical activity. American journal of health promotion : AJHP. 2014;28(3 Suppl):S19-S26.

29. King DK, Litt J, Hale J, Burniece KM, Ross C. 'The park a tree built': Evaluating how a park development project impacted where people play. Urban Forestry & Urban Greening. 2015;14(2):293-9.

30. Smith BJ, MacKenzie-Stewart R, Newton FJ, Haregu TN, Bauman A, Donovan RJ, et al. A longitudinal study examining uptake of new recreation infrastructure by inactive adults. The international journal of behavioral nutrition and physical activity. 2019;16(1):59-.

31. Evenson KR, Cho G-H, Rodríguez DA, Cohen DA. Park use and physical activity among adolescent girls at two time points. Journal of sports sciences. 2018;36(22):2544-50.

32. Sanders T, Feng X, Fahey PP, Lonsdale C, Astell-Burt T. The influence of neighbourhood green space on children's physical activity and screen time: findings from the longitudinal study of Australian children. The international journal of behavioral nutrition and physical activity. 2015;12:126-.

33. Michael YL, Perdue LA, Orwoll ES, Stefanick ML, Marshall LM, Osteoporotic Fractures in Men Study G. Physical activity resources and changes in walking in a cohort of older men. American journal of public health. 2010;100(4):654-60.

34. Dowda M, Saunders RP, Colabianchi N, Dishman RK, McIver KL, Pate RR. Longitudinal associations between psychosocial, home, and neighborhood factors and children's physical activity. Journal of physical activity & health. 2020:1-7.

35. Ng M, Rosenberg M, Thornton A, Lester L, Trost SG, Bai P, et al. The effect of upgrades to childcare outdoor spaces on preschoolers' physical activity: findings from a natural experiment. International journal of environmental research and public health. 2020;17(2):E468.

36. Tannis C, Senerat A, Garg M, Peters D, Rajupet S, Garland E. Improving physical activity among residents of affordable housing: is active design enough? International journal of environmental research and public health. 2019;16(1):151.

## Results

### Study selection process

**Fig. 1:**
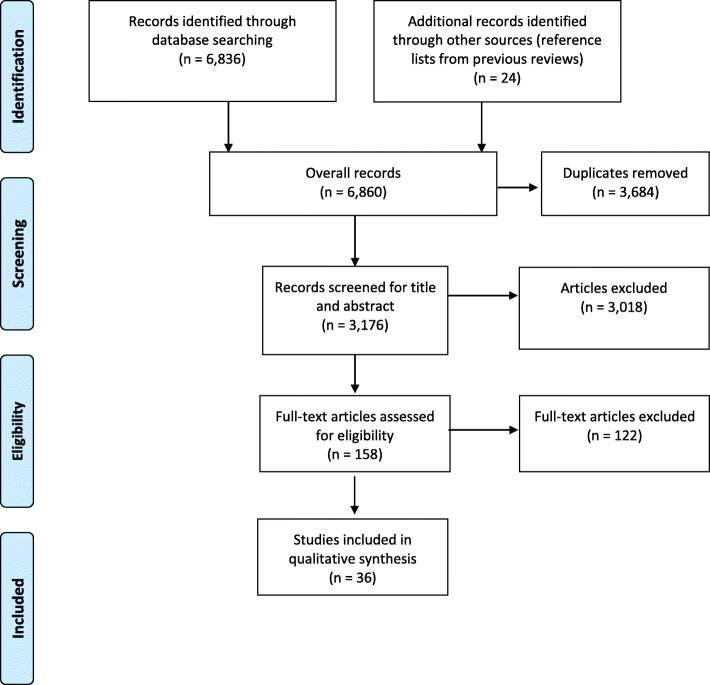
PRISMA flow-diagram of the study selection process

The search across the six databases resulted in 6836 publications (see Fig. 1). In addition, 24 articles were identified by screening reference lists from previous reviews and other relevant publications. Then, 3864 duplicates were removed resulting in 3176 records that were screened. In the first step, 3018 articles were excluded based on titles and abstracts. This left 158 potentially eligible studies for the full-text screening. In total, 36 studies were included for data extraction and methodological quality assessment.

### Characteristics of included studies

From the 36 included studies, more than half were conducted in the USA (*n* = 19), the others took place in Australia (*n* = 8), European countries (*n* = 7), Mexico (*n* = 1), and China (*n* = 1). Eleven of the included studies had prospective longitudinal cohort designs; others were intervention studies with pre-post-measurements (natural and quasi-experiments). Half of the studies only focused on adults, ten focused on children and adolescents, and eight studies on all age groups. Most samples included both men and women except for three studies that included women only and one that included men only. Fourteen studies measured follow-up effects during 1 year or less, and only seven studies followed participants for up to 5 years or longer.

Exposure and outcome measurement varied widely. Sixteen studies used objective methods for measuring PA (accelerometers or observation), fourteen studies measured PA outcomes using self-report questionnaires, and six studies combined subjective and objective measurements. The built environment was measured objectively (with GIS, similar mapping tools or audited by researchers) in 11 studies, improvements or constructions of new infrastructures were used as a measure of the built environment in 25 studies. According to the ecological model of four domains of PA [16], 22 studies were focused on the built environment related to the transportation domain, 17 studies on the built environment related to the recreation domain, and one study on the built environment related to the household domain. We did not find studies that investigated effects of the built environment on occupational PA taking sex/gender into account.

### Risk of bias assessment

The average quality score of included studies was 0.83 with a range between 0.6 and 0.96. In most of the studies, the research question, the objective, the analysis, and the results were sufficiently described and the study design was appropriate. The main sources of bias were related to confounding, measurement, and subject selection. In prospective longitudinal cohort studies, there were high rates of loss to follow-up. Intervention studies suffered from poor sample representativeness or lack of a control group. Quality scores of the individual studies are reported in Table 1.

### Effects of built environmental changes on PA outcomes

Most of the studies (*n* = 27) reported a statistically significant beneficial relationship between at least one built environmental characteristic and PA behavior.

#### Effects of the built environment on males and females

Most of the selected studies showed that built environment characteristics have a similar effect on the PA behavior of males and females.

Fourteen intervention studies and eight longitudinal studies investigated how different characteristics of roads and transport infrastructure influence the residents’ PA. Eleven of these studies assessed the impact of new routes for walking and cycling and showed that all effects were similar for both sexes/genders. There was no positive effect of changes in built environment on overall PA [25, 26]. In one study, the level of PA even decreased after installation of a new trail [27]. However, five studies [28–32] reported that the level of walking and/or cycling increased after built environment interventions and three studies [33–35] reported an increasing number of users of new facilities. The availability of public transport had a significantly positive effect on PA [36] and walking [28, 30, 31, 37]. The movability index (calculated based on residential density, land use mix, street connectivity, availability of public transport, and public open spaces) was associated with higher levels of PA in boys and girls [38]. Landscape diversity [39], distance to school, and daily destinations [40, 41] had no effect on PA. Local road density was negatively associated with PA level [42]. The studies that focused on street network characteristics showed diverse results: three longitudinal studies reported no effect of street connectivity and road environments on PA [39–41], two studies [42, 43] reported positive associations of intersection density with PA levels, and one study [37] with walking for both males and females.

The studies that investigated effects of built environment on recreational PA behavior largely showed a positive effect in both, males and females. Park and green space improvements [44, 45], the creation of new parks [46–48], and the installation of outdoor PA equipment [49, 50] were associated with increased PA and use of facilities. One study [51] reported that after park access improvement, the number of users increased, but overall PA decreased. The construction of a new aquatic and recreation center did not affect overall PA of residents, but the number of users increased 1 year after the construction [52]. In two studies [1, 2], the availability of PA facilities and public open spaces was associated with increased PA and two studies [40, 53] reported no effect.

One study had a specific focus on changes in PA after moving into houses with active design (more attractive stairwells, outdoor community garden area, etc.) [54]. It showed that female and male residents which used to live in houses with an elevator increased their number of steps per day and MVPA after moving in the new environment.

#### Effects of the built environment on females

The review of selected studies also identified a number of built environment characteristics that had a larger/stronger effect on PA behavior in females. Some studies reported that building of new bike lanes [33, 34], availability of public transport [28, 55], and house density [55] increased active transportation and using of PA facilities more in women than in men. The movability index and the distance to daily destinations had a positive effect on the overall PA level in females [53]. Street network characteristics had no effect on the PA level or walking [53, 55]. It was shown that local road density and land use mix were even negatively associated with overall PA and active transportation in females [43, 56]

New recreation facilities such as pocket parks [47] and new aquatic and recreation centers [52] attracted more female users; however, it did not affect their overall level of PA. Moving into houses with active design increased work-related MVPA in women [54].

#### Effects of the built environment determinants on males

The studies that investigated the effects of transport-related built environment on PA have shown that the presence of dead-end roads [41] and intersection connectivity [38] had stronger positive associations with overall PA in boys. In three studies that investigated the effect of building new bike lanes on active travel, one found increases in using facilities in favor of men [29]. Local road density and proportion of local roads was positively associated with PA among men in low urbanicity areas [43]. All other transportation-related built environment characteristics had no positive effect on PA behavior in men and street connectivity was even negatively associated with mean of daily PA in men [53].

The availability of parks and green zones [57, 58], the creation of new pocket parks [47], the installation of outdoor gyms in parks [59], and outdoor PA equipment [50] increased PA levels significantly more in men than in women. Michael et al. [60] reported that in a male sample, the proximity to recreation facilities was associated with a higher likelihood of maintaining or increasing walking time in high-socioeconomic status neighborhoods. After moving into houses with an active design, men had higher levels of moderate recreational PA [54].

## Discussion

This systematic review included 36 studies that examined the determinants of built environmental features associated with PA, taking sex/gender into account. To the best of our knowledge, this is the first study that aims to identify built environmental determinants that are relevant for both males and females, and that could be starting points for intervention programs addressing the needs of both populations. The review summarized results from studies with longitudinal and intervention designs that currently provide the highest level of evidence in this field of research [17]. A variety of built environmental features were identified and allocated to the transport, recreational, or household environment.

The majority of the reviewed studies reported that the built environment determined PA behavior in a similar way in males and females. Creating new infrastructure for walking, cycling, and public transportation showed a positive effect on PA behavior. The findings were most consistent for the availability of public transport, which was positively associated with overall PA and walking. The improvement of walking and cycling infrastructure had no effect on the overall level of PA, but it attracted more users and had a positive effect on active transportation in more than half of the studies. These findings are in line with previous reviews [9, 11, 32]. These types of interventions seem to be promising for promoting active travel. In women, the availability of public transport, specially organized safe cycling lanes, house density, and the distance to daily destinations, showed to be more relevant for PA behavior. In men, street network characteristics and road environment, such as intersection connectivity, local road density, and the presence of dead-end roads, were identified as important determinants of PA. Some of the built environmental determinants of PA identified in women are more related to safety aspects. Similar results were found in previous studies showing that safety concerns were barriers of PA in women [61, 62]. These findings should be taken into account when planning future environmental interventions.

Based on the results of the current review, improvements in recreation facilities can be considered a useful strategy to enhance overall PA, especially in males. The creation of new parks as well as the renovation of existing parks and green areas can make them more attractive for visitors and increase overall PA levels of users independent of sex/gender. Outdoor equipment, playgrounds, and green areas/spaces rather attract male users. No specific characteristics of the built environment related to recreation were found to increase overall PA in females only.

Houses with active design seem to be promising for increasing the daily number of steps and the level of PA. In the reviewed studies, an increase in steps was observed for both sexes/genders: males increased moderate recreational PA and females increased work-related MVPA. However, only one study with a small sample analyzed this type of the built environment [54]; hence, results must be interpreted with caution.

The comparison of the effects of different features of the built environment on PA behavior in males and females shows that environmental interventions should be complex and include improvements both in transport-related and recreational environments to address PA behavior of both genders. Urban planners, policy makers and other practitioners should take into account that easy access to public transport and safety when using cycling lanes is more important for females than for males. For males, it is more important to have playgrounds and outdoor PA equipment in parks.

The methodology, measures, and analytical approaches used to evaluate built environmental determinants of PA behavior are characterized by substantial variability. This finding is in line with previous reviews on similar topics [9, 13] and hampers comparisons between studies and interventions. Even studies that examined similar built environmental characteristics had significant differences in research design and evaluation approaches that resulted in a diverse quality of research. In most studies, the methodological quality of the studies was limited due to a lack of appropriate control conditions, consideration of cofounders, and poor sample representatives (small number of participants, high loss to follow-up).

Blinding was not described in the majority of studies and hardly implemented in the reviewed studies. This limitation can affect results especially in studies that used subjective tools for assessing PA behavior. We excluded questions on blinding during the quality assessment for all studies where it was not applicable, which did not affect the final quality score. However, it is recommended that future studies find a way to prevent bias associated with participants’ self-reports and investigators’ partiality when blinding is not possible.

The timing and number of follow-up measures were inconsistent and varied from 6 months to 15 years. This limits comparison of results and a clear understanding of the impact of the built environment on PA. In almost 40% of the studies, the follow-up period was 1 year or less. In their review of experimental studies, Mayne et al. [63] found that studies with longer follow-up times reported stronger impacts of changes in the built environment on PA. The short follow-up periods in built environmental interventions were usually caused by pragmatic and economic factors. However, longer periods are recommended.

Moreover, it has to be taken into account that almost all of the studies were conducted in the USA, Australia, and Europe, where gender roles for PA behaviors are less pronounced and opportunities for an active lifestyle might be more similar for males and females [64]. This could be one of the explanations why most of the built environmental determinants of PA behavior were similar for males and females in the included studies. The conduction of further studies in developing countries and in Asian countries is necessary to investigate whether sex/gender differences in built environmental determinants of PA are related to culture and socio-economic factors.

Although the number of studies focused on built environmental determinants of PA behavior is growing, most of them do not take sex/gender into account or do not present sex/gender-related results in the articles. Adding such information to the articles could contribute to a better understanding of the issue and confirm or disprove the results of this review. The review showed that the included studies are predominantly related to transportation and recreation environments. This does not allow us to draw conclusions about whether there is a difference in the influence of occupational and household environment on PA in males and females. This could be recommended as one of the topics for future research in this field.

### Strengths and limitations

The strengths of this review lie in the systematic search of relevant studies in six electronic databases and in the evaluation of methodological quality (risk of bias) of all included studies. The analysis of longitudinal and intervention studies allows to identify associations between built environment characteristics and PA, which may prove useful for planning future interventions by focusing on sex/gender differences in effects of the built environment on PA; future interventions can be informed so that they are effective for both males and females. In this manner, inequalities caused by the intervention can be prevented by including different parameters that make it effective both for males and females.

The review also has some limitations. Publication bias cannot be ruled out as only published journal articles in English language were eligible for inclusion and all other types of publications and gray literature as well as articles in other languages were excluded. Differences in the approaches used to examine and report PA outcomes hinder a comprehensive comparison of the reviewed studies. For this reason, a quantitative synthesis of results with meta-analytic procedures could not be conducted. Furthermore, age stratification was not possible due to high heterogeneity of included studies. This limits the interpretation of the results since the effects of the built environment on PA by gender may be different for children and adults. More than half of the studies measured PA behavior of participants with self-reported instruments and, in this case, people may under- or overestimate their level of PA. This, combined with the fact that built environmental intervention studies are rarely blind for participants, can affect the results and make them less reliable.

## Conclusions

This review sheds light on the relevance of the built environment on PA. The findings from this review support the hypothesis that designing an activity-friendly environment can have a positive effect on PA. In general, improvements are beneficial for both genders. However, it shows that in order to address both genders equally, urban planning should focus on built environmental characteristics related to different PA domains as some of them are more effective PA in females and some in males. The focus on sex/gender differences introduces a new aspect that should be further analyzed in future research and considered by urban planners and other practitioners.

## Abbreviations

GIS: Geographical information systems
MVPA: Moderate to vigorous physical activity
PA: Physical activity
WHO: World health organisation
